# Adverse drug reactions in hospitals: population estimates for Portugal and the ICD-9-CM to ICD-10-CM crosswalk

**DOI:** 10.1186/s12913-023-10225-z

**Published:** 2023-11-08

**Authors:** Raquel Ascenção, Paulo Nogueira, Filipa Sampaio, Adriana Henriques, Andreia Costa

**Affiliations:** 1 Laboratório de Farmacologia Clínica e Terapêutica, Faculdade de Medicina, Universidade de Lisboa, Avenida Professor Egas Moniz, 1649-028 Lisboa, Portugal; 2 Escola Nacional de Saúde Pública - Universidade Nova de Lisboa, Lisboa, Portugal; 3 Department of Public Health and Caring Sciences, Uppsala University, Uppsala, Sweden; 4 Nursing Research, Innovation and Development Centre of Lisbon (CIDNUR), Nursing School of Lisbon, Lisboa, Portugal; 5 Instituto de Saúde Ambiental (ISAMB), Faculdade de Medicina, Universidade de Lisboa, Lisboa, Portugal

**Keywords:** Drug-related side effects and adverse reactions, Poisoning, Medical errors, Hospital information systems, International classification of diseases, Portugal

## Abstract

**Background:**

Adverse drug reactions (ADR), both preventable and non-preventable, are frequent and pose a significant burden. This study aimed to produce up-to-date estimates for ADR rates in hospitals, in Portugal, from 2010 to 2018. In addition, it explores possible pitfalls when crosswalking between ICD-9-CM and ICD-10-CM code sets for ADR identification.

**Methods:**

The Portuguese Hospital Morbidity Database was used to identify hospital episodes (outpatient or inpatient) with at least one ICD code of ADR. Since the study period spanned from 2010 to 2018, both ICD-9-CM and ICD-10-CM codes based on previously published studies were used to define episodes. This was an exploratory study, and descriptive statistics were used to provide ADR rates and summarise episode features for the full period (2010–2018) as well as for the ICD-9-CM (2010–2016) and ICD -10-CM (2017–2018) eras.

**Results:**

Between 2010 and 2018, ADR occurred in 162,985 hospital episodes, corresponding to 1.00% of the total number of episodes during the same period. Higher rates were seen in the oldest age groups. In the same period, the mean annual rate of episodes related to ADR was 174.2/100,000 population. The episode rate (per 100,000 population) was generally higher in males, except in young adults (aged '15–20', '25–30' and '30–35' years), although the overall frequency of ADR in hospital episodes was higher in females.

**Conclusions:**

Despite the ICD-10-CM transition, administrative health data in Portugal remain a feasible source for producing up-to-date estimates on ADR in hospitals. There is a need for future research to identify target recipients for preventive interventions and improve medication safety practices in Portugal.

**Supplementary Information:**

The online version contains supplementary material available at 10.1186/s12913-023-10225-z.

## Background

Noxious and unintended effects resulting from the use of medicinal products are frequent and pose a significant burden to the patient and the healthcare system, as well as to the healthcare professional, the regulator, and the industry [1].

Common terms to report the adverse effects of medication use in medication safety literature are adverse drug events (ADE), adverse drug reactions (ADR) and medication errors, despite a lack of homogeneity between terms and definitions [2].

The current ADR definition endorsed by the European Medicines Agency (EMA) covers both adverse outcomes resulting from the authorised use of a medicinal product at normal doses and those resulting from medication errors and uses outside the terms of the marketing authorisation [2, 3].

Coding of patient records using the International Classification of Diseases (ICD) is commonly employed to classify medication harm in hospitals [2].

In Portugal, previously published studies on ADR in hospitals have addressed the 2000–2015 period and used different sets of ICD – 9th revision – Clinical Modification (ICD-9-CM) codes for episode selection [4–7]. Scriparu et al. found that ADR were present in 1.46% of all discharges in the 2004–2013 period [6]. The mean age of affected patients was 63.79 years, being 54.50% female [6]. Despite being more frequent in women in absolute terms, the rate of ADR was higher in men (1.48 vs 1.44%) [6]. No estimate was provided for the rate per 100,000 population, as in other published papers on the Portuguese population [4–7].

More recently, ICD-10-CM has come into force in Portugal [8], with profound implications for health outcomes coding [9]. Previous studies have advised the need to investigate the impact of the transition from ICD‐9‐CM to ICD‐10‐CM codes on health outcomes on a case‐by‐case basis [9].

This study aimed to produce up-to-date estimates for ADR rates in hospitals, in Portugal, from 2010 to 2018. In addition, we aimed to explore possible pitfalls when crosswalking between ICD-9-CM and ICD-10-CM code sets for medication harm in administrative hospital databases.

## Methods

### Terminology and definitions

The ADR definition followed the EMA recommendation, encompassing adverse effects resulting from medication use, including medication errors [3, 10].

Hospital episode refers to either outpatient or inpatient episodes, as long as it was included in the Portuguese Hospital Morbidity Database.

### Study setting

In 2020, Portugal registered a population of 10,295,909 inhabitants [11]. Life expectancy is higher than the European average, despite a drop of 0.8 years between 2019 and 2020 due to COVID-19 [11].

The Portuguese National Health Service (NHS) is a tax-financed health system providing universal access to high-quality care [11]. The NHS coexists with two other systems: the health subsystems, special health insurance schemes that provide coverage for particular professions or sectors, and private voluntary health insurance schemes [11]. Around one-fourth of the population has a second health insurance coverage [12].

### Data sources

The Portuguese Hospital Morbidity Database was used for this study. This database gathers information on inpatient, surgical or day hospital outpatient episodes in public hospitals nationwide. Emergency Department visits are not included in the database, except when they result in a subsequent inpatient admission. Despite its primary administrative purpose, the Hospital National Morbidity Database is also frequently used for clinical or health services research. It comprises patient demographics, clinical information (ICD diagnosis and procedures codes, episode severity, in-hospital mortality), admission and discharge information (date, place) and diagnostic-related grouping codes (for cost information). Patient records are linked with an encrypted, unique patient identification code. The transition from ICD-9-CM to ICD-10-CM/PCS in Portugal started in August 2016 [13]. The ICD-10-CM/PCS transition period was heterogeneous among hospitals due to differences in prior training and adaptation of information systems [8]. Consequently, ICD-9-CM clinical coding was still present in some facilities even after the mandatory deadline of January 2017 [8].

Portuguese resident population estimates were obtained from Statistics Portugal [14].

### Episode identification

This study included all episodes related to ADR registered in the Hospital Morbidity Database between 2010 and 2018. Episodes related to ADR were defined as episodes (inpatient or outpatient) with at least one ICD code of ADR. As such, ADR could be present on admission (as a cause for admission) or occurring during the hospital episode as a nosocomial event.

Since the study period spanned from 2010 to 2018, both ICD-9-CM and ICD-10-CM were used to define episodes. Both code sets were identified based on previously published studies.

The ICD-9-CM code set relied on E-codes, as previously studied in the Portuguese setting [5, 6]. E-codes are supplemental to the ICD-9-CM diagnosis codes and used to describe external causes of injury and poisoning codes, including other categories other than ADR, including transport accidents or accidental falls [15, 16].

Under ICD-10-CM, E-codes are absent, and diagnosis codes T36-T50 ('Poisoning by, adverse effect of and underdosing of drugs, medicaments and biological substances') provide information on both the substances involved and intent of injuries related to drugs ('1': accidental, '2': intentional self-harm, '3': assault, '4': undetermined, '5': adverse effect and '6': underdosing) [17, 18]. Due to the greater specificity of the ICD-10-CM compared to the ICD-9-CM, we revised the T36-T50 codes to prevent problems due to backward mapping [19]. Codes T36-T50 (ICD-10-CM) were crosswalked to ICD-9-CM (backward mapping) using the General Equivalence Mappings (GEM) method. We followed the General Equivalence Mappings (GEM) endorsed by the Administration of the Health System (ACSS) in Portugal, originally developed by the Center for Medicare and Medicaid Services and the Centers for Disease Control and Prevention. ACSS is the entity responsible for managing the Portuguese Hospital Morbidity Database [13]. Other authors have previously recommended manual refinement when conducting studies using ICD-9-CM-based algorithms mapped to ICD-10-CM codes [20].

Table 1 provides a summary of all ICD codes.

**Table 1:** ADR definition according to the ICD-9-CM and ICD-10-CM

ICD-9-CM	ICD-10-CM
E930-E949.9 E850-E858.9	T36 to T50 and a - sixth character of '5' (adverse event), '1' (accidental) or '6' (underdosing) or - sixth digit of 'X' and a fifth character of '5' (adverse event), '1' (accidental) or '6' (underdosing)

### Statistical analyses

For the study period (2010 to 2018), irrespective of the ICD code set in use, the total number of episodes related to ADR was summarised by age group, sex, health region, admission circumstances and discharge options. Annual crude rates were computed by age group and sex, based on the number of episodes with an ICD code of ADR relative to the resident population in Portugal. We assumed that the age distribution of the population covered by NHS hospitals (the population at risk) was comparable to that of the general population. It was necessary because around one-fourth of the population has a second health insurance coverage.

To explore possible pitfalls when crosswalking between ICD-9-CM and ICD-10-CM code sets, we analysed the characteristics of episodes from the 2010–2016 period (ICD-9-CM coded episodes) compared to those from the 2017–2018 period (ICD-10-CM coded episodes).

Data were analysed using R Version 4.2.1 and R Studio Server Version 2022.02.3 Build 492.

## Results

### ADR in hospitals: total episodes and rates

Between 2010 and 2018, there were 162,985 episodes related to ADR, from a total of 16,375,364 hospital episodes (1.00%).

In the same period, the mean annual rate of episodes related to ADR was 174.2/100,000 population. Table 2 reports the annual number of episodes related to ADR, as well as the annual rate for the same period.

**Table 2:** The annual number and rates of episodes related to ADR

Year	Episodes			Population			Episode rate ^a^		
	**M**	**F**	**Total**	**M**	**F**	**Total**	**M**	**F**	**Total**
2010	6,083	7,470	13,553	5,053,543	5,519,178	10,572,721	120.4	135.3	128.2
2011	7,429	9,050	16,479	5,030,437	5,511,961	10,542,398	147.7	164.2	156.3
2012	8,072	9,259	17,331	4,995,697	5,491,592	10,487,289	161.6	168.6	165.3
2013	9,016	10,527	19,543	4,958,020	5,469,281	10,427,301	181.8	192.5	187.4
2014	9,442	10,861	20,303	4,923,666	5,451,156	10,374,822	191.8	199.2	195.7
2015	9,863	11,327	21,191	4,901,509	5,439,821	10,341,330	201.2	208.2	204.9
2016	9,386	10,545	19,931	4,882,456	5,427,117	10,309,573	192.2	194.3	193.3
2017	8,163	9,481	17,644	4,867,692	5,423,335	10,291,027	167.7	174.8	171.5
2018	7,822	9,188	17,010	4,852,366	5,424,251	10,276,617	161.2	169.4	165.5

*F* Females, *M* Males

^a^Rate per 100,000 population at risk (estimated resident population for Portugal)

A total of 33,348 patients had more than 1 episode during the study period. The mean number of episodes per patient was 1.26, which was higher among the younger age groups (0–20 years) (Supplementary Appendix, Table S1).

### ADR in hospitals: episodes and rates by age group and sex

**Fig. 1:**
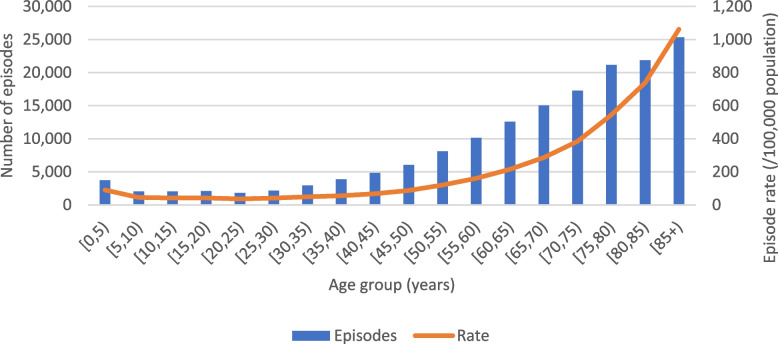
Number and rate of ADR-related episodes, per age group, between 2010 and 2018

The number and rate of episodes related to ADR were higher in women (Table 2) and increased with advancing age after a drop from '0–5' to '5–10' years (Fig. 1).

Considering the distribution by sex and age group, however, the episode rate was generally higher in men, except for young adults ('15–20', '25–30' to '30–35' years) (Table 3).

**Table 3:** Distribution of ADR-related episodes and rates, between 2010 and 2018

Age groups (years)	Episodes			Population			Episode rate^a^		
	**M**	**F**	**Total**	**M**	**F**	**Total**	**M**	**F**	**Total**
0–5	1,952	1,764	3,716	2,099,393	2,004,989	4,104,382	93.0	88.0	90.5
5–10	1,150	883	2,033	2,308,309	2,202,375	4,510,684	49.8	40.1	45.1
10–15	1,142	884	2,026	2,483,506	2,366,507	4,850,013	46.0	37.4	41.8
15–20	1,028	1,066	2,094	2,555,769	2,448,497	5,004,266	40.2	43.5	41.8
20–25	921	899	1,820	2,531,072	2,481,552	5,012,624	36.4	36.2	36.3
25–30	1,040	1,115	2,155	2,622,834	2,642,593	5,265,427	39.7	42.2	40.9
30–35	1,350	1,587	2,937	2,942,058	3,087,519	6,029,577	45.9	51.4	48.7
35–40	1,890	1,975	3,865	3,364,706	3,603,756	6,968,462	56.2	54.8	55.5
40–45	2,404	2,443	4,847	3,449,588	3,710,444	7,160,032	69.7	65.8	67.7
45–50	3,050	3,012	6,063	3,316,279	3,595,950	6,912,229	92.0	83.8	87.7
50–55	4,142	3,943	8,085	3,210,570	3,517,355	6,727,925	129.0	112.1	120.2
55–60	5,416	4,704	10,120	2,975,582	3,309,496	6,285,078	182.0	142.1	161.0
60–65	6,779	5,787	12,566	2,724,730	3,101,002	5,825,732	248.8	186.6	215.7
65–70	7,845	7,169	15,014	2,392,718	2,852,586	5,245,304	327.9	251.3	286.2
70–75	8,683	8,582	17,265	1,971,213	2,518,396	4,489,609	440.5	340.8	384.6
75–80	9,524	11,638	21,162	1,620,271	2,264,592	3,884,863	587.8	513.9	544.7
80–85	8,805	13,056	21,861	1,135,065	1,823,388	2,958,453	775.7	716.0	738.9
≥ 85	8,155	17,201	25,356	761,723	1,626,695	2,388,418	1,070.6	1,057.4	1,061.6

*F* Females, *M* Males

^a^Rate per 100,000 population at risk (estimated resident population for Portugal)

### ADR in hospitals: episodes and rates by geographical area

By geographical area of residence, the Lisbon and Tagus Valley health region registered the highest rate over the years (206.9/100,000 population) (Table 4). The autonomous regions of Azores and Madeira registered rates of 45.3 and 40.5, respectively (not displayed in Table 4).

**Table 4:** Distribution of ADR-related episodes and rates by geographical area of residence, between 2010 and 2018

Area of residence	Episodes	Population	Episode rate^a^
Lisbon and Tagus Valley	52,542	25,398,930	206.9
Centre	38,410	20,440,118	187.9
Alentejo	11,163	6,598,373	169.2
North	51,800	32,650,164	158.7
Algarve	5,681	3,987,539	142.5

^a^Rate per 100,000 population at risk (estimated resident population for Portugal)

### ADR in hospitals: episodes by admission circumstances and discharge options

Between 2010 and 2018, most ADR-related hospital episodes were inpatient (*n* = 161,778; 99.3%) rather than outpatient (*n* = 1,207; 0.7%) admissions. The majority were urgent admissions (*n* = 134,099; 82.3%), and patients were discharged home (*n* = 138,808; 85.2%). Most were classified as medical cases (*n* = 143,868; 88.3%) (Supplementary Appendix, Table S2).

### ADR in hospitals: in-hospital mortality

During the 2010–2018 period, 14,289 patients died during an ADR-related hospital episode (8.8% in-hospital mortality) (Supplementary Appendix, Table S2).

### Summary of main features of ADR in hospitals

Table 5 summarises the main features of ADR in hospitals during the 2010–2018 period, as well as for the 2010–2016 (ICD-9-CM coded) and 2017–2018 (ICD-10-CM) periods.

**Table 5:** Summary of main features of ADR in hospitals during the 2010–2018 period

	**2010**	**2011**	**2012**	**2013**	**2014**	**2015**	**2016**	**2017**	**2018**	**ICD-9-CM period**	**ICD-10-CM period**
										**2010–2016**	**2017–2018**
**Number**	13,553	16,479	17,331	19,543	20,303	21,191	19,931	17,644	17,010	18,333	17,327
**% of all episodes**	0.69	0.78	0.87	1.15	1.18	1.21	1.11	1.03	1.06	1.00	1.04
**Inpatient, %**	99.56	99.79	99.59	99.61	99.73	99.42	98.53	98.79	98.35	99.46	99.35
**Male, %**	44.88	45.08	46.58	46.13	46.51	46.54	47.09	46.27	45.98	46.12	46.12
**Age, %**											
0–19 years	6.91	6.54	6.85	5.16	5.52	5.99	6.87	5.64	5.32	6.26	5.48
20–39 years	7.59	7.28	7.20	6.64	6.57	5.91	6.05	6.44	6.34	6.75	6.39
40–64 years	26.87	27.39	26.50	25.74	24.97	24.48	24.91	25.36	24.74	25.84	25.05
≥ 65 years	58.63	58.79	59.46	62.46	62.95	63.63	62.17	62.56	63.60	61.16	63.08
**In-hospital mortality, %**	8.04	7.76	8.48	8.84	8.72	9.46	9.61	8.73	8.77	8.70	8.75
**Urgent, %**	85.17	84.03	82.06	82.88	81.26	82.77	80.02	79.94	83.47	82.60	81.71

## Discussion

### Summary of main findings

This study aimed to produce up-to-date estimates for coded ADR rates in hospitals, in Portugal, from 2010 to 2018. By presenting the features of ADR in hospitals over the years, we sought to illustrate possible pitfalls when crosswalking between ICD-9-CM and ICD-10-CM code sets for medication harm in administrative hospital databases.

Between 2010 and 2018, the frequency of ADR in hospital episodes was 1.00%, and the mean annual rate of episodes related to ADR was 174.2/100,000 population. These are in line with previous estimates from a recent systematic review of studies with national coverage conducted in Europe, Central Asia, East Asia and the Pacific, North America, Latin America and the Caribbean. The relative frequency of ADE-related hospitalisations ranged from 0.03% to 7.3% and from 9.7 to 383.0/100,000 population [21]. The ADE definition considered by the review authors is similar to the ADR definition used in our study, and the included studies also focused on ADE as the cause of hospital admission and/or occurring during inpatient treatment. However, the review authors found heterogeneity concerning database scope, ADE definition, and ICD codes in the included studies [21].

In contrast, Stausberg estimated a higher overall prevalence rate (and 95% confidence interval, CI) of coded ADR in hospitals in 2006. This was estimated at 3.22% (3.20%–3.23%) for England, 4.78% (4.73%–4.83%) for Germany, and 5.64% (5.63%–5.66%) for the USA [22]. However, the ICD code sets used for the analysis were remarkably different from the ones used in our study and may explain these differences. Carrasco-Garrido et al., using slightly different ICD-9-CM E-codes, estimated that 1.69% of all acute admission in Spain during 2001–2006 had an ADR present (either as the cause or occurring during the hospital episode) [23].

It should be noted that we included all hospital episodes present in the Hospital Morbidity Database, irrespective of episode type (inpatient or outpatient) and the primary diagnosis (i.e., whether ADR was the cause for seeking care or not). This is not the case in other cited studies focused on inpatient episodes. The ratio of inpatient to outpatient episodes (in each country) is expected to impact the estimated frequency of ADR in hospital episodes. However, since 99,3% of included episodes in our study were classified as inpatients, including outpatient episodes is not expected to impact the estimated rates per 100,000 population. Nonetheless, criteria such as different admission criteria, differences in prescribing patterns and population characteristics may also play a role in the wide variation reported between studies conducted in different countries.

In our study, the absolute number and rate of ADR in hospitals were higher in women. However, in further analysis considering the episode rate per 100,000 population, by sex and age group, the episode rate was generally higher in men. Therefore, our results may reflect the Portuguese population age pyramid and the profile of patients subject to hospital care and are unadjusted for medication use and other confounding variables. The female gender has been associated with hospital admissions attributed to ADR [24] and sex has long been recognised as an important determinant of drug use and response [25] in previous studies.

As expected, the rate of ADR in hospitals was higher in older adults because of the increased risk of ADR likely due to multimorbidity and polypharmacy, as well as altered pharmacokinetics and pharmacodynamics [26].

Lisbon and Tagus Valley Health Region had the highest episode rate. From a public health standpoint, it would be helpful to explore the features of these episodes to ascertain if this result would persist after adjusted analyses for medication use and other confounding variables. Were the same results to persist, it would be relevant to ascertain whether they were due to a true high rate in that particular Health Region or rather due to the miscoding of ADRs.

In our study, in-hospital mortality was estimated at around 8.8%, in line with previous national and international studies. Scripcaru et al. estimated an 8.0% in-hospital mortality rate between 2004–2013 in Portugal [6]. This rate was higher compared to non-ADR hospital episodes and length of stay, in line with similar studies [22]. However, this might be due to unadjusted comparisons. In another study conducted by Sousa-Pinto et al., between 2000 and 2015, in Portugal, in-hospital mortality was found to be lower for ADE hospitalisations of all studied categories (poisoning, ADR and late effects) after propensity score matching [7]. However, this association was only observed for medical episodes and urgent admissions, not for surgical and planned admissions. Episode characteristics can, by themselves, hinder the conclusions as they also affect the outcome. For example, severe urgent admissions are prone to higher and early in-hospital mortality, irrespective of the ADR status. Residual confounding may persist despite the use of statistical procedures aimed to eliminate bias [27].

The current study proposes a set of ICD-10-CM codes to capture ADR in hospitals and compares the results with those obtained with a set of ICD-9-CM codes previously reported in the literature. Our study is the first to report ADR rates in hospitals using ICD-9-CM and ICD-10-CM in the same report.

The characteristics of hospital episodes identified through ICD-9-CM and ICD-10-CM were similar.

Cheng et al. previously studied the validity of ICD-10-CM T codes for ADR identification in hospital claims data [28]. Inpatient episodes with T codes in a primary or secondary diagnosis were identified, and pharmacists performed a retrospective review of the medical chart to confirm the ADR [28]. The positive predictive value for a T code representing an ADR was 57% [28]. It notes to be mentioned that even so, the use of T codes increased the ADR reporting rate by 9.17% [28]. However, contrary to our study, it seems that no manual refinement took place, and intentional self-harm, assault, and undetermined circumstances were included for each of the 'Poisoning' categories.

### Strengths and limitations

To the best of our knowledge, this is the first study in Portugal to estimate the annual rate of episodes related to ADR per 100,000 population after the uptake of ICD-10-CM.

However, some limitations are worth mentioning. In our study, it was not possible to distinguish between ADR present on admission and those occurring during the hospital episode. A previous study in Portugal focused exclusively on ADR acquired during the hospital episode between 2013–2015 through the application of "present at admission" coding [5]. Around 28.5% of all ADR occurred during the hospital stay, i.e., were not present on admission. Our study did not have access to the "present at admission" variable.

Our study only examined one set of ICD-10-CM codes, which can be perceived as a limitation. Previous studies suggested testing multiple ICD‐10‐CM outcome definitions as part of sensitivity analysis when using data from both the ICD‐9‐CM and ICD‐10‐CM eras [9].

The mapping method applied in this work relied fundamentally upon backward mapping (ICD-10-CM to ICD-9-CM), as GEM from ICD-9-CM to ICD-10-CM (forward mapping) suggested no codes. The obtained ICD-10-CM codes were then subject to manual refinement. The combination of tentative forward mapping, backward mapping, and manual refinement does not conflict with the results and recommendations of other authors [29]. However, it is not risk-free, as some areas (such as sequela identification or underdosing coding in ICD-10-CM) may be mapped outside E-codes or have no translation in ICD-9-CM, and therefore not captured in our analysis. This situation might have been minimised by the iterative application of the forward and backward mapping strategies [29] or the dismissal of sequela and underdosing T codes, but all these solutions would also require manual refinement at the expense of subjective decisions. We opted for this more straightforward, easily replicable strategy that may be subject to validation in further studies.

Furthermore, E-Codes (ICD-9-CM), crosswalked to T codes (ICD-10-CM) may also be criticised. It is worth mentioning that using the same database (albeit for a single Institution), followed by a chart review for validation, Miguel et al. [16] found that E-codes generated 284 signals for ADR, with 95% positive predictive value. Six diagnostic codes (without simultaneous E-coding) queries were selected and generated an additional 87 signals, with 87.6% positive predictive value [16]. This is to be expected as E-codes should always be accompanied by an appropriate diagnosis that conveys the clinical presentation (E-codes are merely the description of the external cause). A similar rationale applies to the ICD-10-CM when considering the crosswalked T codes. Therefore, if coded right, a diagnosis for ADR should always be accompanied by an E-code or diagnosis T code. On another note, extending into additional diagnosis codes would make the crosswalk from ICD-9-CM to ICD-10-CM increasingly difficult and prone to gaps. In addition, the exploratory nature of our study precluded an in-depth analysis of the clinical characteristics of patients and hospital episodes. The current study provides, however, important evidence to guide further studies, particularly for the ICD-10-CM coding period.

There are specific limitations concerning the database used for analysis. The Portuguese Hospital Morbidity Database covers around 70% of all inpatient hospital episodes in Portugal [30]. It was created to monitor hospital productivity for mainland Portugal within the publicly financed NHS, explaining the underrepresentation of the islands of Madeira and Azores within the database. Therefore, the results presented may better represent the setting of mainland Portugal. Moreover, administrative databases pose relevant challenges, such as the low sensitivity for the detection of ADR, as extensively discussed by other authors [16, 31, 32]. Low sensitivity may result from undercoding. Undercoding underestimates the frequency of ADR and may result from the absence of information in the discharge summary narrative text (either because of underdiagnosis or underreporting) or solely from the underrecognition by the coding physician during the encoding procedure [33].

### Implications for future research

It would be crucial to test additional code sets [9] and to evaluate the performance of the proposed ICD-10-CM codes to capture true positive ADR in hospitals in Portugal, as previously conducted for ICD-9-CM code sets [16]. Furthermore, examining key comorbidities and drug classes would help identify population groups at higher risk of ADR, especially those preventable, such as those arising from accidental poisoning and underdosing, thus contributing to designing appropriate public health interventions. A recent review of reviews acknowledged that various factors, including but not limited to event detection methods, patient age and setting, can influence estimates of preventable ADR [34]. This reinforces the need to consider using local data when designing context appropriate public health interventions. As an example, the National Action Plan for Adverse Drug Event Prevention, developed by the Office of Disease Prevention and Health Promotion (U.S. Department of Health and Human Services), used data from a nationally representative sample of hospital admissions for ADE among older adults to select the preventable targets for intervention. Three key targets were identified, namely, anticoagulants, insulin, and oral diabetes agents, and the basis for initiatives such as surveillance and prevention [35]. Similar initiatives could be devised in the Portuguese context.

Our work also sheds light on some areas, such as repeat ADR, which are beyond the scope of the present work, hence were not fully addressed, and should be subject to further research.

## Conclusions

This study shows that the crosswalk between ICD-9-CM and ICD-10-CM is possible and supports the sustained use of administrative health data in Portugal for producing up-to-date estimates of ADR in hospitals. Future research should explore the use of ICD-10-CM coded data to identify target recipients for preventive interventions and contribute to improving medication safety practices in Portugal.

### Supplementary Information

**Additional file 1: Table S1.** Distribution of episodes and patients, per age group, between 2010 and 2018. **Table S2.** The annual number of ADR-related hospital episodes by admission status, episode class and discharge options.

## Data Availability

All available data are within the manuscript and its supporting information file. This study was based on the analysis of proprietary data owned by the Portuguese Central Administration of the Health System, I.P. (ACSS). Aggregated data can be accessed via the Transparency Portal held by the Portuguese Ministry of Health at the following link: https://transparencia.sns.gov.pt/explore/?sort=title&q=morbilidade+hospital.

## Abbreviations

ADR: Adverse drug reactions
EMA: European Medicines Agency
GEM: General Equivalence Mappings
ICD: International Classification of Diseases
ICD-9-CM: ICD – 9th revision – Clinical Modification
ICD-10-CM: ICD – 10th revision – Clinical Modification
NHS: National Health Service
