# Supplementary material for: Adverse drug reactions in hospitals: population estimates for Portugal and the ICD-9-CM to ICD-10-CM crosswalk
Source: BMC Health Serv Res. 2023 Nov 8;23:1222. doi: 10.1186/s12913-023-10225-z (PMC10634004; doi:10.1186/s12913-023-10225-z)
Supplement: Supplementary file 1 — Additional file 1: Table S1. Distribution of episodes and patients, per age group, between 2010 and 2018. Table S2. The annual number of ADR-related hospital episodes by admission status, episode class and discharge options. [file 12913_2023_10225_MOESM1_ESM.docx]

**Supplementary information**

Table S1. Distribution of episodes and patients, per age group, between 2010 and 2018.

| **Age Group** | **Episodes** | **Patients** | **Mean number of episodes per patient** |
| --- | --- | --- | --- |
| **[0,5)** | 3,716 | 2,223 | 1.67 |
| **[5,10)** | 2,033 | 1,012 | 2.01 |
| **[10,15)** | 2,026 | 927 | 2.19 |
| **[15,20)** | 2,094 | 1,297 | 1.61 |
| **[20,25)** | 1,820 | 1,381 | 1.32 |
| **[25,30)** | 2,155 | 1,590 | 1.36 |
| **[30,35)** | 2,937 | 2,269 | 1.29 |
| **[35,40)** | 3,865 | 2,959 | 1.31 |
| **[40,45)** | 4,847 | 3,823 | 1.27 |
| **[45,50)** | 6,063 | 4,812 | 1.26 |
| **[50,55)** | 8,085 | 6,321 | 1.28 |
| **[55,60)** | 10,120 | 8,027 | 1.26 |
| **[60,65)** | 12,566 | 9,857 | 1.27 |
| **[65,70)** | 15,014 | 12,327 | 1.22 |
| **[70,75)** | 17,265 | 14,566 | 1.19 |
| **[75,80)** | 21,162 | 18,194 | 1.16 |
| **[80,85)** | 21,861 | 19,262 | 1.13 |
| **[85,120)** | 25,356 | 22,348 | 1.13 |

Table S2. The annual number of ADR-related hospital episodes by admission status, episode class and discharge options.

| **Year** | **Admission status** | | | **Episode class** | | | **Discharge options** | | |
| --- | --- | --- | --- | --- | --- | --- | --- | --- | --- |
|  | **Urgent** | **Elective** | **Other** | **Medical** | **Surgical** | **Other** | **Home** | **Deceased** | **Other** |
| **2010** | 11,543  (85.2) | 1,988  (17.4) | 22  (0.2) | 11,852  (87.4) | 1,200  (8.9) | 501  (3.7) | 11,857  (87.5) | 1,090  (8.0) | 606  (4.5) |
| **2011** | 13,847  (84.0) | 2,614  (15.9) | 18  (0.1) | 14,568  (88.4) | 1,525  (9.3) | 386  (2.3) | 14,276  (86.6) | 1,279  (7.8) | 924  (5.6) |
| **2012** | 14,222  (82.1) | 3,088  (17.8) | 21  (0.1) | 15,488  (89.4) | 1,777  (10.3) | 66  (0.4) | 14,863  (85.8) | 1,469  (8.5) | 999  (5.7) |
| **2013** | 16,197  (82.9) | 3,321  (17.0) | 25  (0.1) | 17,443  (89.3) | 2,100  (10.7) | 0  (0.0) | 16,572  (84.8) | 1,728  (8.8) | 1,243  (6.4) |
| **2014** | 16,498  (81.3) | 3,774  (18.6) | 31  (0.1) | 18,058  (88.9) | 2,245  (11.1) | 0  (0.0) | 17,270  (85.1) | 1,770  (8.7) | 1,263  (6.1) |
| **2015** | 17,540  (82.8) | 3,596  (17.0) | 55  (0.2) | 18,836  (88.9) | 2,353  (11.1) | 2  (0.0) | 17,826  (84.1) | 2,004  (9.5) | 1,361  (6.4) |
| **2016** | 15,948  (80.0) | 3,938  (19.8) | 45  (0.2) | 17,600  (88.3) | 2,324  (11.7) | 7  (0.0) | 16,739  (84.0) | 1,916  (9.6) | 1,276  (6.4) |
| **2017** | 14,105  (79.9) | 3,538  (20.1) | 1  (0.0) | 15,199  (86.1) | 2,445  (13.9) | 0  (0.0) | 14,968  (84.8) | 1,541  (8.7) | 1,135  (6.3) |
| **2018** | 14,199  (83.5) | 2,811  (16.5) | 0  (0.0) | 14,824  (87.1) | 2,184  (12.8) | 2  (0.0) | 14,437  (84.9) | 1,492  (8.8) | 1,081  (6.4) |
| **2010-2018** | 134,099  (82.3) | 28,668  (17.6) | 218  (0.1) | 143,868  (88.3) | 18,153  (11.1) | 964  (0.6) | 138,808  (85.2) | 14,289  (8.8) | 9,888  (6.1) |
